# Treatment of Active Tuberculosis in Chicago, 2008-2011: The Role of Public Health Departments

**DOI:** 10.1371/journal.pone.0164162

**Published:** 2016-10-12

**Authors:** Reid Fletcher, Joshua D. Jones, Neha S. Shah

**Affiliations:** 1 Northwestern University, Feinberg School of Medicine, Chicago, Illinois, United States of America; 2 Chicago Department of Public Health, Chicago, Illinois, United States of America; 3 Centers for Disease Control and Prevention, Atlanta, Georgia, United States of America; University of Minnesota, UNITED STATES

## Abstract

**Objective:**

Evaluate differences in TB outcomes among different provider types in Chicago, IL.

**Methods:**

We retrospectively reviewed all TB cases reported to the Chicago Department of Public Health (CDPH) from 2008 through 2011. Provider type was stratified into three groups: public, public-private, and private providers. Multivariate regression was used to evaluate treatment duration and time to sputum culture conversion. A Cox proportional hazard model was used to assess treatment completion.

**Results:**

Of 703 cases, 203 (28.9%), 314 (44.7%), and 186 (26.5%) were treated by public, public-private and private providers, respectively. Adjusted regression showed private provider patients had a 48-day (95% CI 22.0–74.3) increase in treatment duration and a 30-day (95% C.I. 9.5–51.1) increase in time to sputum culture conversion. Cox model showed increased risk of remaining on treatment was associated with extra-pulmonary TB (aHR 0.78, 95% C.I. 0.62–0.98), being foreign-born (aHR 0.74, 95% C.I. 0.58–0.95), and any drug resistance (aHR 0.59, 95% C.I. 0.46–0.76). There were no differences in outcomes between public and public-private providers.

**Conclusion:**

Patients treated solely in the private sector had prolonged time to sputum culture conversion and treatment duration which lead to increased cost for treatment, prolonged infectiousness, potential for transmission, and the possibility for increased medication side effects.

## Introduction

In the 1900s, tuberculosis (TB) was the second leading cause of death in the United States.^1^ Due to better social and living conditions, improved public health infrastructure, and the development of effective antibiotics, TB incidence declined dramatically.[1] As TB rates declined, TB funding also declined, with elimination of federal TB funding in 1972. The shrinking of public health infrastructure, the HIV epidemic, rising homelessness urban overcrowding, emerging TB drug resistance, and an influx of immigrants from TB-endemic countries led to a TB resurgence in the mid-1980’s and a subsequent refunding of TB control.[2,3] Since the 1993 peak in TB cases, the incidence of TB has fallen to an historic low and funding for TB control is again at risk.[4,5]

Similar to national TB trends, from 1993 to 2011, TB rates in Chicago declined nearly 79%, from the recent peak of approximately 29 cases per 100,000 in 1993 to just over 6 cases per 100,000 in 2011.[6] Historically, publicly funded programs have managed TB care, treatment and the public health response to each active TB case.[7] However in 2011, as TB incidence reached a historic low, the City of Chicago considered closing TB clinics operated by the City.[5] The closure of City-operated TB clinics shifted medical care of TB patients to safety net clinics and private providers.

Current literature suggests that outcomes in TB patients treated by public health providers versus other providers may be different. Patients treated by non-public health providers are less likely to receive directly observed therapy (DOT), less likely to undergo HIV testing, less likely to have documented sputum-culture conversion, and are more likely to be prescribed inappropriate treatment regimens and to die during treatment.[8–14] In 2011, TB patients in Chicago were cared for by three different types of providers: clinics operated by the Chicago Department of Public Health (CDPH, “public providers”), clinics and hospitals without affiliation with CDPH (“private providers”), and safety net clinics who received some TB funding from CDPH (“public-private providers”). We conducted a retrospective chart review of Chicago TB cases to assess differences in TB care by provider type.

## Methods

### Data Collection

**Fig 1:**
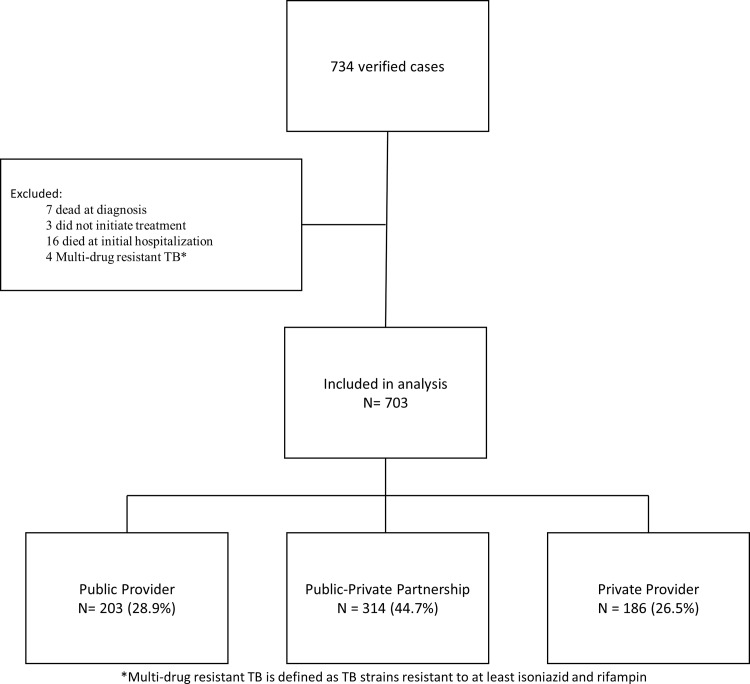
Number of tuberculosis case-patients eligible for inclusion, Chicago, IL, 2008–2011.

As required by state regulation, cases of active TB were reported to the CDPH TB Control Program using a standardized case report form that collects data on socio-demographics, clinical characteristics, and treatment outcomes.^15^ All cases reported from January 1, 2008 through December 31, 2011 were included in our analysis. Cases were excluded if they were dead at the time of TB diagnosis, lost to follow-up prior to treatment initiation, diagnosed with multi-drug resistant (MDR) TB, or died prior to initiating outpatient TB treatment (Fig 1). MDR TB cases (defined by initial resistance to isoniazid [INH] and rifampin [RIF]) were excluded, as management of MDR TB patients often requires multiple care settings and specialized expertise which defies simple categorization of provider type.

Data were abstracted from the Illinois National Electronic Disease Surveillance System (I-NEDSS), a CDPH internal clinical management database, and medical records. This analysis was conducted as part of a program evaluation of the CDPH TB Control program which, as the Local Health Authority under Illinois law, is mandated to routinely collect and analyze surveillance data for public health purposes. As a program evaluation, the study was exempted from IRB review by Centers for Disease Control and Prevention (CDC), CDPH and Northwestern University.

### Definitions

Providers were stratified into three categories: public, private and public-private partnership. Public providers were defined as clinics operated by CDPH, at which TB care was provided without cost to the patient. Public-private partnership providers treated TB patients in medical safety net clinics that received partial funding from CDPH, and worked closely with the CDPH TB control program, including participating in monthly TB case review conferences. Private providers were defined as any other outpatient provider without funding from or affiliation with CDPH; such providers are required to provide TB care updates to CDPH. The two primary outcomes were based on routine indicators of quality TB care used by the CDC Division of TB Elimination: completion of TB treatment within 365 days and sputum culture conversion to negative within 60 days after initiation of TB treatment. [15] Treatment duration was the time from treatment initiation to treatment completion or discontinuation, including death and loss to follow-up. Analysis of time to sputum culture conversion was restricted to pulmonary or combined pulmonary/extra-pulmonary cases who had an initial positive sputum culture and documented sputum culture conversion. Cases without a documented date of sputum culture conversion were excluded from this portion of the analysis. Time elapsed to sputum culture conversion was calculated from TB treatment initiation to collection date of at least one negative sputum culture with no subsequent positive.[16] Having ever received directly observed therapy (DOT) was defined as either DOT for the entire duration of therapy or a combination of DOT and self-administered therapy. Any substance abuse was defined has having used injection drugs, non-injection drugs or excess alcohol in the last 12 months.[16] Drug resistance was defined as resistance to any of the first-line anti-TB medications–isoniazid, rifampin, ethambutol and pyrazinamide.

### Statistical Analysis

Chi-square tests or Fischer’s exact test, t-test or analysis of variance (ANOVA) were used to compare differences in patients by provider type. To evaluate characteristics associated with treatment duration in patients completing treatment, multivariate linear regression was constructed using variables statistically significant in univariate analysis at p<0.05 as well as clinically significant variables. An adjusted Cox proportional hazard model (aHR) evaluated treatment duration in all patients including those who died or were lost to follow-up. We constructed multivariate linear regression assessing characteristics associated with days to sputum culture conversion using variables significant in univariate analysis.

Time-to-treatment completion was analyzed using Kaplan-Meier analysis and the log-rank test was used to assess for statistically significant differences. All analyses were conducted using Stata version 12 (College Station, TX).

## Results

### Univariate Analysis

Of 734 cases reported to CDPH from 2008–2011, 703 (95.8%) met inclusion criteria. Of these, 203 (28.9%), 314 (44.7%), and 186 (26.5%) were treated by public providers, public-private providers and private providers, respectively (Fig 1). In univariate analysis, compared to public providers, private providers were significantly more likely to have patients who were older than 65 years of age (29.3% versus 15.5% for private and public, respectively), who had extra-pulmonary or synchronous extra-pulmonary and pulmonary TB (51.6% versus 22.8% for private and public, respectively), be living with HIV (8.1% versus 4.4% for private and public, respectively), and who died during the TB treatment compared to public or public-private providers (8.5% versus 2.4% for private and public, respectively, Table 1). Additionally, compared to public providers, private provider patients were significantly less likely to have ever received DOT (38.8% and 96.1% for private and public, respectively), had longer median days on treatment (231.5 versus 210 for private and public, respectively), had a lower rate of treatment completion (87.2% versus 94.2% for private and public, respectively) to complete treatment within one year (79.8% versus 91.7% for private and public, respectively), to have sputum culture conversion within 60 days of treatment initiation (38.3% versus 61.1% and for private and public, respectively, Table 1). Public-private providers patients differed in racial/ethnic background with more white Hispanic patients, were less likely to have any drug resistance (8.2% versus 20.1% for public-private and public, respectively), less likely to receive DOT (89.2% versus 96.1% for public-private and public, respectively), and more likely to be lost or move prior to treatment completion or refuse therapy (6.7% versus 3.4% for public-private and public, respectively).

**Table 1:** Socio-demographic, clinical characteristics and treatment outcomes of TB cases, Chicago, 2008–2011.

Characteristic	Provider type, n (%)				
	Public (N = 203)	Public-Private (N = 314)	*P* value^1^	Private (N = 186)	*P* value^2^
Age			0.361		0.0002
0–18 years	14 (6.8)	13 (4.1)		15 (8.0)	
19–45 years	89 (44.2)	142 (45.1)		62 (33.5)	
46–65 years	69 (33.5)	121 (38.7)		55 (29.3)	
>65	31 (15.5)	38 (12.1)		54 (29.3)	
Male	121 (59.6)	209 (66.6)	0.45	92 (49.5)	0.108
Race/ethnicity			<0.0005		0.383
White, non-Hispanic	19 (9.4)	29 (9.2)		30 (16.4)	
White, Hispanic	41 (20.2)	117 (37.1)		37 (20.2)	
African-American/black, non-Hispanic	83 (40.9)	121 (38.7)		68 (36.2)	
Asian	59 (29.1)	46 (14.6)		50 (26.6)	
Foreign-born	115 (57.3)	191 (60.6)	0.345	97 (52.7)	0.373
Any substance abuse, alcohol or drugs			0.147^3^		<0.0005
No	149 (73.8)	213 (67.9)		165 (88.3)	
Yes	54 (26.2)	97 (30.8)		20 (11.2)	
Unknown	0	4 (1.3)		1 (0.5)	
Homeless within the past year			0.004		0.105
No	186 (91.6)	260 (82.8)		178 (95.7)	
Yes	17 (8.4)	54 (17.2)		8 (4.3)	
Site of disease			0.157		<0.0005
Pulmonary	156 (77.2)	218 (69.5)		90 (48.4)	
Extrapulmonary	29 (14.1)	64 (20.3)		70 (37.8)	
Both	18 (8.7)	32 (10.2)		26 (13.8)	
Chest X-Ray			0.456		<0.0005
Abnormal	155 (76.2)	225 (71.7)		90 (48.4)	
Normal	42 (20.9)	80 (25.4)		84 (45.1)	
Missing/Not done	6 (2.9)	9 (2.9)		12 (6.5)	
Cavitary chest x-ray	69 (44.4)	82 (39.2)	0.078	27 (28.0)	<0.0005
HIV status			0.027		0.004
Negative	177 (87.2)	247 (78.7)		139 (74.7)	
Positive	9 (4.4)	37 (11.7)		15 (8.1)	
Not Known	17 (8.4)	30 (9.6)		32 (11.2)	
Any drug resistance present^4^	34 (20.1)	21 (8.2)	0.002	20 (11.8)	0.036
Ever received DOT	195 (96.1)	280 (89.2)	0.005	72 (38.8)	<0.0005
Sputum conversion documented	95 (75.4)	120 (75.0)	0.997	60 (75.9)	0.963
Sputum conversion within 60 days of treatment initiation	58 (61.1)	63 (52.5)	0.209	23 (38.3)	0.006
Median days on treatment^5^	210	202	0.305	231.5	0.038
Completed treatment ever	193 (94.2)	292 (92.7)	0.338	163 (87.2)	0.009
Reason for stopping therapy			0.002^3^		0.019^3^
Completed treatment	193 (94.2)	292 (92.7)		163 (87.2)	
Died	5 (2.4)	0		16 (8.5)	
Lost/Moved/Refused	5 (3.4)	21 (6.7)		6 (3.2)	
Provider decision to stop therapy	0	1 (0.3)		1 (0.5)	
Completed treatment within 365 days of treatment initiation	187 (91.7)	293 (93.3)	0.607	149 (79.8)	0.001

1 P value indicates a comparison between public-private and public providers.

2 P value indicates a comparison between public and private providers.

3 Fischer’s exact test due to small sample size.

4 Multi-drug resistance is excluded from the analysis. Drug resistance here is defined as any other drug resistance pattern which does not meet criteria for MDR.

5 Moody’s median test was used to compare median treatment durations.

### Multivariable analysis

Patients of private providers had a 48.1 day increase in duration of treatment compared to public providers (95% Confidence Interval [CI] 21.9–74.3; Table 2). There was no significant difference between public and public-private providers (-4.82 days, 95% CI: -24.09, 14.45). Increased duration of therapy was associated with extra-pulmonary TB (30.7 days, 95% C.I. 9.85–51.6), being born outside of the United States (25.5 days, 95% C.I. 2.48–48.6), any substance abuse (24.2 days, 95% C.I. 3.34–45.03) and resistance to any first-line anti-TB drug (54.65 days, 95% C.I. 31.2–78.07).

**Table 2:** Multivariate Linear Regression Predicting Days on Treatment. Chicago, 2008–2011.

Characteristic	Coefficient	95% Confidence Interval
Provider type		
Public (ref)	1.00	
Public-Private	-4.82	-24.09, 14.45
Private	48.14	21.98, 74.30
Ever received DOT	19.58	-5.67, 44.84
Site of disease		
Pulmonary (ref)	1.00	
Extra-Pulmonary	30.72	9.85, 51.60
Both	41.74	16.55, 66.93
HIV positive	3.57	-4.13, 11.27
Age at TB diagnosis	-0.06 (per year)	-0.49, 0.37
Female	-9.99	-27.41, 7.43
Race/Ethnicity		
White, non-Hispanic (ref)	1.00	
White, Hispanic	16.53	-13.47, 46.53
African-American, non- Hispanic	23.27	-6.42, 52.97
African-American, Hispanic	-36.81	-168.80, 95.18
Asian	-9.94	-40.42, 20.53
Foreign born	25.55	2.48, 48.63
Any substance use, alcohol or drugs^1^	24.18	3.34, 45.03
Any drug resistance^2^	54.65	31.22, 78.07
Constant	184.59	

TB = tuberculosis, HIV = human immunodeficiency virus, DOT = directly observed therapy.

1 Defined has having used injection drugs, non-injection drugs or excess alcohol in the last 12 months.

2 Multi-drug resistance (MDR) is excluded from the analysis. Drug resistance here is defined as any other drug resistance pattern which does not meet criteria for MDR.

Patients of private providers had a 30.3 day increase in documented time to sputum culture conversion (95% C.I. 9.48–51.12; Table 3). The difference between public and public-private providers was not significant (8.42 days, 95% CI: -4.66, 21.50). A history of substance abuse (either alcohol or illicit drugs) was associated with an increased time to sputum culture conversion (18.74 days, 95% C.I. 4.63–32.84). Female gender was associated with a 16.99 day decrease in time to sputum culture conversion (95% C.I. -4.28, -29.7).

**Table 3:** Multivariate Linear Regression Predicting Days to Sputum Culture Conversion Among 275 Patients With Documented Sputum Culture Conversion. Chicago, 2008–2011.

Characteristic	Coefficient	95% Confidence Interval
Provider Type		
Public (ref)	1.00	
Public-Private	8.42	-4.66, 21.50
Private	30.3	9.48, 51.12
Ever received DOT	-0.622	-23.69, 22.45
Cavitary chest x-ray	1.35	-10.36, 13.06
Age at TB diagnosis	-0.042 (per year of age)	-0.38, 0.29
Female	-16.99	-29.7, -4.28
Race/Ethnicity		
White, non-Hispanic (ref)	1.00	
White, Hispanic	-2.93	-23.78, 17.93
African-American, non- Hispanic	-6.05	-24.27, 12.16
African-American, Hispanic	2.16	-67.09, 71.40
Asian	-3.38	-24.26, 17.51
Homelessness^1^	6.69	-11.98, 25.36
Any substance use, alcohol or drugs^2^	18.74	4.63, 32.84
Any drug resistance^3^	-1.2	-17.28, 14.88
Constant	64.93	

TB = tuberculosis, HIV = human immunodeficiency virus, DOT = directly observed therapy.

1 Defined as homelessness at any time within the past 12 months.

2 Defined has having used injection drugs, non-injection drugs or excess alcohol in the last 12 months.

3 Multi-drug resistance (MDR) is excluded from the analysis. Drug resistance here is defined as any other drug resistance pattern which does not meet criteria for MDR.

### Kaplan-Meier and Cox proportional hazard analysis

**Fig 2:**
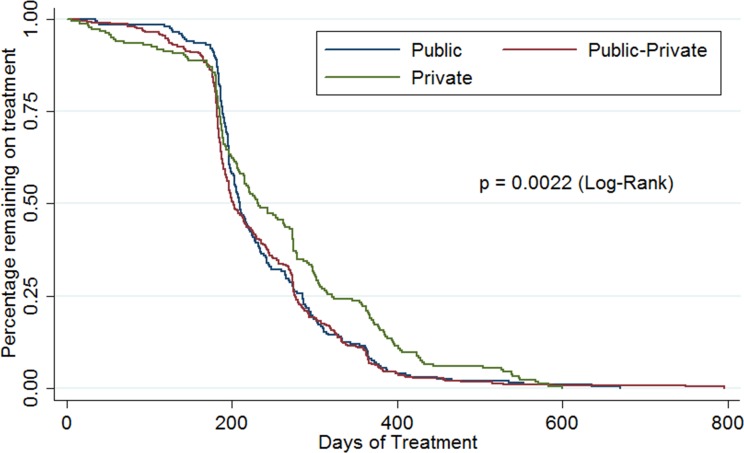
Kaplan-Meier Curve for the prportion of patients remaining on treatment by provider type, Chicago 2008–2011.

Fig 2 shows the Kaplan-Meier analysis for the likelihood of a patient remaining on treatment, stratified by provider type. A vertical line marks 365 days of treatment. There was a significant difference (log-rank test, p = 0.0022) for the probability of remaining on treatment at a given time point during the treatment course by provider type. In stratified analysis, the probability of remaining on treatment at a given time point during the treatment course between public and public-private providers (log rank test, p = 0.44) was not significant. However, there were significant differences between public and private providers (log rank tests, p = 0.0149) and between public-private and private providers (log rank test, p = 0.0007). Kaplan-Meier curves stratified by age groups were also created and there was a consistent significant difference between public and private providers and between public-private and private providers across all age groups.

In the adjusted Cox proportional hazard model, multiple variables were independently associated with an increased risk of remaining on treatment, including the presence of extra-pulmonary TB (aHR 0.78, 95% C.I. 0.62–0.98), being born outside of the United States (aHR 0.74, 95% C.I. 0.58–0.95), and any drug resistance pattern (aHR 0.59, 95% C.I. 0.46–0.76). (Table 4)

**Table 4:** Multivariate Hazard Ratios for Remaining on Tuberculosis Treatment. Chicago Department of Public Health, 2008–2011.

Characteristic	Adjusted Hazard Ratio	95% Confidence Interval
Provider Type		
Public (ref)	1.00	
Public-Private	1.02	0.83, 1.26
Private	0.70	0.53, 0.92
Ever received DOT	0.94	0.72, 1.24
Site of disease		
Pulmonary (ref)	1.00	
Extra-Pulmonary	0.78	0.62, 0.98
Both	0.71	0.54, 0.93
HIV positive	1.00	0.92, 1.12
Age at TB diagnosis	1.00 (per year of age)	1.00, 1.01
Female	1.07	0.88, 1.29
Race/Ethnicity		
White, non-Hispanic (ref)	1.00	
White, Hispanic	0.82	0.60, 1.12
African-American, non-Hispanic	0.75	0.55, 1.02
African-American, Hispanic	2.15	0.76, 8.98
Asian	1.05	0.76, 1.45
Foreign born	0.74	0.58, 0.95
Any substance use, alcohol or drugs^1^	0.80	0.64, 1.00
Any drug resistance^2^	0.59	0.46, 0.76

TB = tuberculosis, HIV = human immunodeficiency virus, DOT = directly observed therapy.

1 Defined has having used injection drugs, non-injection drugs or excess alcohol in the last 12 months.

2 Multi-drug resistance (MDR) is excluded from the analysis. Drug resistance here is defined as any other drug resistance pattern which does not meet criteria for MDR.

## Discussion

Our study evaluated differences in TB treatment outcomes between public and private providers. Patients managed by private provider were less likely to meet CDC outcome targets for timely treatment completion within one year. CDC guidelines recommend that cases of both pulmonary and extra-pulmonary TB should complete treatment within 12 months. The findings that these patients had a 48-day longer treatment duration as well as overall lower treatment completion and treatment completion within one year suggests that private providers patients are remaining on treatment longer than recommended by CDC guidelines. Unfortunately, we did not have data to assess why private provider patients received prolonged treatment. Additionally, we do not have data on subsequent outcomes of TB treatment (e.g. recurrence rates of active TB, long term adverse effects of TB treatment), however, prolonged treatment duration increases patient exposure to toxic medications (associated with poor outcomes and increased mortality[17,18]) and adds economic burden to the health system and the patient’s household.[19] There are reasons why treatment is prolonged beyond one year, including regimen changes due to side effects, missed appointments, and acquired resistance. Though our study was not able to determine why providers chose to prolong therapy, the univariate analysis showed private provider patients are less likely to receive DOT which allows for health care workers to assess medication side effects and barriers to care on a daily basis, ensure treatment adherence and minimize treatment interruptions.

We found that private provider patients had a 30-day longer time to sputum-culture conversion when compared with public provider patients. Sputum culture positivity is a proxy for infectiousness, and the timely conversion of sputum culture is an indicator of the efficacy of therapy.[20] An increased time to sputum-culture conversion leads to a prolonged infectious period and the potential for greater TB transmission in the community. Longer time to documented sputum culture conversion could indicate higher bacterial burden at the initiation of treatment, non-standard or inadequate treatment during the treatment initiation phase, non-adherence with the treatment regimen, or delays in the collection of sputum to document conversion. The decreased use of DOT among private provider patients could represent the possibility of less robust treatment during the induction phase resulting in a longer time to conversion. Additionally the prolonged time to sputum conversion by private provider patients could be related to an increased lag time in the collection of sputum samples due to less frequent patient contact by private provider patients not utilizing DOT.

Our study did not find significant differences between public and public-private providers. It is well documented that the direct input of public health departments in TB care impacts the quality of TB care, perhaps because of TB-specific expertise.[21] As funding for the direct provision of TB care by public health departments decreases, our observation that public and public-private providers seem to provide similar quality TB care suggests a possible mechanism for continued publicly-funded TB control program involvement in the provision of TB care. In Chicago, these partnerships allow for close collaboration between the City’s TB experts and safety net providers, who are frequently geographically closer to where patients live and may have services beyond just TB care. Collaborating closely with community clinics, CDPH continues to provide DOT services with the added benefits of timely sputum collection, medical assessments in the field, and mitigation of barriers to care such as rides to appointments.

The univariate analysis demonstrated a statistically significant increase in mortality rate in the private provider group (8.5%) compared to the public (2.4%) and public-private (0%) provider groups. The private provider group also had significantly more patients over 65 years of age However age-stratified Kaplan-Meier analysis showed that patients of private providers were more likely to remain on treatment across all age groups suggesting that the increased mortality rate is not the primary driver of prolong treatment duration.

Our analysis may be limited by differences in data quality between public providers and private providers; our experience was that data collection from private providers was more challenging because these providers were not directly accountable to CDPH. There is also an unmeasured bias introduced in a patient’s choice, or absence of choice, of provider based on their insurance status and geographic location. Our results may not be generalizable as TB programs are unique across the country and many do not have publicly-funded TB clinics. Lastly, while a survival analysis is the most appropriate statistical method to analyze data such as these with censored events, we also included a linear regression. This regression is likely to be influenced by the censored events, however, it was included in the study because it allows us to quantify duration of treatment across groups.

Previous studies have also documented less favorable outcomes in TB care provided by private physicians.[8–13] As public funding for TB control is re-evaluated and the Patient Protection and Affordable Care Act (PPACA) expands opportunities for patients to obtain health insurance, we expect a shift of TB care to the private sector [22]. With continuing decline in TB cases, maintaining expertise in TB treatment and being aware of current national standards can be challenging for private physicians. Over the past 20 years the rate of TB has fallen over 75% in Chicago limiting the potential number of TB patients any single provider may treat. In contrast, CDPH TB providers oversee the care of 100–200 cases per year. Additionally, a central public tenet of TB control is to minimize the economic barriers to access of TB care. Although the PPACA has improved health care coverage, there are no provisions that guarantee coverage for TB diagnosis and treatment without cost-sharing and approximately 40–50 million people remain uninsured.[23,24] This uninsured population is likely to have disproportionately more people at risk for tuberculosis than the insured population due to the exclusion of undocumented persons, who are at increased risk of TB from exposure in their country of origin, from the insurance markets.[25] The limited TB expertise in the private sector and challenges with the PPACA, highlight the need for continued public health funding of TB control activities. Our study suggests a public-private collaboration where TB programs work with community providers to case manage and provide expert consultation is a viable option and allows for treatment outcomes consistent with national standards.
